# The match between motivation and performance management of health sector workers in Mali

**DOI:** 10.1186/1478-4491-4-2

**Published:** 2006-02-09

**Authors:** Marjolein Dieleman, Jurrien Toonen, Hamadassalia Touré, Tim Martineau

**Affiliations:** 1 KIT Development, Policy and Practice, Royal Tropical Institute, Amsterdam, The Netherlands; 2 Health advisor, UNICEF Mali, Bamako, Mali; 3 International Health Research Group, Liverpool School of Tropical Medicine, Liverpool, UK

## Abstract

Human resources for health (HRH) play a central role in improving accessibility to services and quality of care. Their motivation influences this. In Mali, operational research was conducted to identify the match between motivation and the range and use of performance management activities.

**Objectives:**

To describe the factors motivating and demotivating health workers in Mali and match the motivators with the implementation of performance management.

**Methods:**

First an exploratory qualitative study was conducted: 28 interviews and eight group discussions were held. This was followed by a cross-sectional survey, during which 370 health workers were interviewed. The study population consisted of health workers of eight professional groups. The following issues were investigated:

• motivating and demotivating factors;

• experiences with performance management, including: job descriptions, continuous education, supervision, performance appraisal and career development.

**Findings:**

The study showed that the main motivators of health workers were related to responsibility, training and recognition, next to salary. These can be influenced by performance management (job descriptions, supervisions, continuous education and performance appraisal). Performance management is not optimally implemented in Mali, as job descriptions were not present or were inappropriate; only 13% of interviewees received 4× per year supervision, and training needs were not analysed. Some 48% of the interviewees knew their performance had been appraised in the last two years; the appraisals were perceived as subjective. No other methods were in place to show recognition. The results enabled the research team to propose adaptations or improvements upon existing performance management.

**Conclusion:**

The results showed the importance of adapting or improving upon performance management strategies to influence staff motivation. This can be done by matching performance management activities to motivators identified by operational research.

## Introduction

A motivated and qualified workforce is crucial to increase the productivity and quality of health services in order to contribute to achieving health services targets. Priority programmes have a stake in a skilled and motivated workforce, as they are implemented primarily by a health facility's existing health staff. Motivation in the work context is defined as "an individual's degree of willingness to exert and maintain an effort towards organisational goals" [1]. The challenge for managers is how to create this kind of motivation. Research has shown that workers and their managers often perceive motivation differently [2]. In addition, little is known about the motivational factors that are important for health workers in resource-poor settings [1,3].

While there are many theories on motivation [1], two different areas of motivation are often confused: motivation to be in a job and motivation to perform. Both are important, and managers need to understand the impact of their activities on both areas. Herzberg's two-factor theory of motivation at the workplace [4] is used in this article to explain the distinction between these two areas of motivation. It distinguishes satisfiers, which are the main causes for job satisfaction (or motivation to perform), from dissatisfiers, which are the main causes for job dissatisfaction (or demotivation to remain in a job) when absent or perceived as insufficient. Examples of motivating factors are achievement, recognition, responsibility and the work itself. Dissatisfiers include: working conditions, salary, relationship with colleagues, administrative supervision, etc. [4].

An organization needs to influence satisfiers through performance management – the "measuring, monitoring and enhancing the performance of staff" [5] – using a range of human resources management (HRM) tools such as: job descriptions, supervision, performance appraisals, continuous education, rewards and career development [5,6]. However, performance management is often underdeveloped in the public health sector in resource-poor settings and published studies are limited, often focusing only on certain aspects of performance management, such as supervision [7-10].

Looking to improve staff performance, the Ministry of Health in Mali in 2001 used operational research to identify motivating factors among their health workers and to determine whether the existing performance management activities were appropriately implemented. The aim was to identify opportunities for improvement of HR activities implemented by managers within the facilities, and the study had therefore a managerial focus, as opposed to a political focus in which power and interests are analysed. This paper presents the results of this research and provides a recommendation for Mali and general lessons learnt for health services and priority-programme managers in other countries.

## Background on Mali

Mali is a low-income country in West Africa (GDP per capita of USD 240) with a population of approximately 10.6 million [11]. Mali is subdivided into seven regions and the capital district of Bamako. The district is the focal point for organization of service delivery.

According to the Ministry of Health, in 2001 Mali had 5173 health workers, of whom the majority (77%) work in the public sector at district, regional and national level. They are managed and paid by the Ministry of Health and are civil servants. Community health centre staff (18% of the workforce) are managed and paid by local health committees, though technical supervision and training is provided by the district teams. Only 5% of the health workforce is employed full-time in private clinics, though dual working is common.

### Research methodology

The main study questions of this operational research, conducted in the context of a broader human resources for health (HRH) situational analysis, were:

1. What motivates and what demotivates health workers?

2. Which performance management activities are used and how much, and how are they perceived by health workers and their managers?

3. How do these performance management activities match with motivating factors?

First an exploratory qualitative study was conducted among managers and health workers. In-depth interviews and group discussions were conducted, in which open questions were asked to identify the range of motivating and demotivating factors and to explore perceptions on performance management, addressing study questions 1 and 2. Health workers were recruited from eight selected health professional groups in the capital and in a district in one rural area, Sikasso. All were employed in the public sector or at community level. Managers of the health facilities visited and two managers at national level were interviewed.

In order to assure trustworthiness of data, sources and methods were triangulated by interviewing health workers and their managers at district, regional and central level and community health centre committees. Interviews were recorded, taped and immediately transcribed. Twenty-eight individual interviews were held with 12 health workers, 13 managers and 3 village committee members. In addition eight group discussions were conducted: four with health workers from teams working at commune level and four with health workers, working at district level. Data were manually analysed using data compilation matrices per respondent group, describing the data per study question. Quality of data collection was assured through providing confidentiality and through the interviewers – experienced researchers – who developed the research and conducted the interviews.

The results of this qualitative study were used to design a cross-sectional and descriptive survey for health workers. The survey consisted of interviews using a questionnaire with two components:

• a scoring table on the importance of motivating and demotivating factors, addressing study question 1. The factors to be scored were derived from the qualitative study.

• closed questions to identify the range and extent of use of performance management activities, addressing study question 2. The selection of variables was based on commonly used HRM tools and the results of the qualitative study – for example: pre-service and in-service training, supervision and performance appraisal.

The survey concentrated on eight professional groups at community and district level: public health doctors, auxiliary nurses, public health nurses, registered nurses, midwives, laboratory technicians, community development workers and the sanitary technicians.

A three-step sampling method was used to recruit respondents. Three out of the seven regions were selected according to the geographical preferences of the HRH: the capital, one remote region and one with relatively easy access: Bamako and the rural areas of Mopti and Sikasso. In each region two districts were randomly selected for the study. In these districts one hospital and two health centres were randomly selected, in which health workers who were present and belonged to the eight professional groups were interviewed.

The number of respondents in each region was based upon the proportion of professionals working in a region with low, medium or high concentration of health workers. When a health centre did not have the number of professionals required for the interviews, a neighbouring centre was selected, until the total number of respondents was achieved.

The interviews were conducted by a team of eight, with a research background and who were not health workers, in order to avoid bias in data collection. The data were analysed using SPSS software. The quality of data collection was assured by guaranteeing anonymity of the interviewees, training and supervision of interviewers by an experienced researcher and by pre-testing the questionnaire. These mechanisms aimed, among other considerations, to avoid bias and socially acceptable answers

Lastly, the results of the survey were triangulated with the results from the interviews and group discussions.

This operational research was carried out within a limited timeframe and budget in order to provide HRH managers and policymakers with quite rapid evidence for decision-making. Pre-testing was not entirely rigorous, resulting in inconsistent interpretation of two motivation-related variables: "training" and "management". "Training" is especially problematic, as it is unclear to what extent training, for which health workers often receive an allowance, is perceived as income generation or as an opportunity to upgrade knowledge and skills [12]. Management is a wide concept: for instance, some health workers perceive "reporting" or "administration" as management, whereas others do not consider these as management activities. Some caution is therefore needed in interpreting the results relating to these two variables.

This article examines the results combining professional groups and levels. Whenever there was a significant difference in results among professionals or type of institution, these have been highlighted. Data are not disaggregated for the private sector, due to the small numbers of staff employed. Even if these numbers were greater, triangulation would not be useful, as it is likely that there is variation in the HR policies and activities of the different private sector employers.

## Findings

### Study population

In the qualitative study, 72 people were interviewed: 51 men and 21 women. Most interviewees at district and regional level in Sikasso were between 45 and 52 years of age and in Bamako they were on average 40 years old. At the community level in both districts respondents were between 28 and 33 years old.

The details of the study population of the survey (N = 370) are shown in Table 1. They were representative for the eight professional groups in the selected study areas. As most health workers are employed in Bamako, the majority of the sample was recruited from Bamako.

**Table 1:** Study population of the survey (N = 370)

**Characteristics**	**Number**	**%**
**Location**		
Bamako	222	60%
Sikasso	115	31%
Mopti	33	9%
**Employer**		
Public sector	274	74%
Community level	71	19%
Private sector	24	6.5%
Missing	1	0.5%
**Type of facility**		
Referral health centre	133	36%
Tertiary hospital in Bamako	89	24%
Community health centre	85	23%
Regional hospital	33	9%
Private sector	30	8%
**Sex**		
Women	207	56% women, overrepresentation of women in Bamako (69% of interviewees)
Men	162	43.5%
Missing	1	0.5%
**Age**		
25–39	185	50%
>39	185	50%

Source: Health worker survey (2001)

Note: Due to the limited information of the human resources management system disaggregated data on the available staff could not be given.

### Motivating and demotivating factors

This section answers study question 1 and describes what motivates and what demotivates health workers. The average scores for motivating and demotivating factors are given in rank order in Tables 2 and 3, all groups combined.

**Table 2:** Average score of factors motivating health workers (N = 367)

**Factor**	**Average score**
To feel responsible	5.7
To increase salary	3.5
To receive training	3.2
To be held responsible	2.6
To be appreciated	2.3
To receive recognition	2.2
To receive promotion	1.5
To receive incentives	1.5
To work within a team spirit	1.3
To receive financial benefits from user fees	0.9
To have your partner living near the workplace	0.7
To have good colleagues	0.7
Others	0.7

Source: Survey for health workers (2001)

Note: The most important factor scored 10 points and the remaining scored 8, 6, 4 and 2 points according to priority. If health workers had scored randomly, the average for each factor would have been 2.3 (SD = 0.17). "Feeling responsible" relates in this table to an internal feeling, whereas "be held responsible", "be appreciated" and "receive recognition" are related to actions by superiors, colleagues and patients.

**Table 3:** Average score of demotivating factors by health workers (N = 354)

**Factor**	**Average score**
Lack of material	8.2
Lack of recognition	3.2
Difficult living conditions	2.9
Lack of a job description	2.5
Subjective performance appraisal	2.5
Poor management	1.8
Partner living far away	1.8
Poor functioning of the health committee	1.2
Living far away from an urban centre	0.5
Living far away from places where decisions are being made	0.4

Source: Survey for health workers(2001)

Note: Each respondent scored the 5 most important factors at respectively 10, 8, 6, 4 and 2 points. If health workers had scored randomly, the average for each factor would have been 2.3 (SD = 0.17).

The results show that apart from salaries, issues related to responsibility, training and recognition scored above average for health workers. Two factors showed a significant difference between the groups. "Feeling responsible" received a significant higher score by physicians (average score 7.6), compared to registered nurses (score 4.8) (p = 0.0025) and "increase in salary" was significantly more motivating for auxiliary nurses and midwives (average score 4.6) compared to physicians (average score 1.6). Health workers and managers said during the in-depth interviews that they were especially encouraged by getting results from their work, being useful to society and taking care of people. When the different types of facilities were compared, the four most important motivating factors were the same for all levels.

Overall health workers complained about the lack of material and equipment. For example, 42% mentioned the lack of a blood-pressure machine and 28% lacked bandages and delivery kits. There were no significant differences between the professional groups. In the qualitative study, health workers and managers at all levels mentioned lack of equipment and lack of recognition as demotivating. Staff at community level complained about poor management: for example they were not allowed to take leave, and rules and regulations were not always clear.

### Performance management activities in Mali

This section presents the experiences of health workers with performance management activities, addressing study question 2. Although salaries were mentioned as the second most important motivating factor, they are not included in the analysis, as the majority of the respondents were public sector employees. Their salaries are set by central government; adjusting levels of pay is beyond the scope of managers at institutional levels.

### Job descriptions

Sixty three percent of the respondents knew what their current tasks should be. In the qualitative study, no one at the lower levels was able to show his or her job description, but most interviewees were convinced of its importance. The existing job descriptions were related to professions and not to posts, which means that a nurse in the hospital has the same job description as a nurse in a community health centre. Not all respondents were trained for the tasks they conduct. For example, auxiliary and registered nurses spent 20% of their time on management tasks, whereas 52% and 38% respectively had had no specific training in management.

### Continuous education

An average of 22% of interviewed health staff had received in-service training in the previous year. This was greater for physicians (28%) and less for auxiliary nurses (14%) and community development workers (7%). Of those who did receive training, 50% attended more than one course. The average number of days in training was 13, which is about 7% of annual working days. Most courses (93%) were organized by priority programmes.

The majority of the respondents highly appreciated training opportunities. However, they also mentioned that in-service training to meet needs at district level often cannot be provided, due to limited resources. Managers had difficulty integrating nationally organized training into their work plans due to poor planning and communication.

Eighty percent of respondents who participated in training were selected by their managers. Health workers responded in the qualitative study that they did not find the selection criteria transparent.

### Supervision and performance appraisal

Each health facility should in principle receive four supervision visits a year. Only 13% had received four visits in the previous year, and 40% received an average of two supervisions per year. The highest rate of supervision was at the community health centre level, where 49% received three or four visits. When asked about the content of supervision visits, at all levels mainly technical topics were mentioned, such as curative consultations and hygiene; planning and management received hardly any attention.

The in-depth interviews showed that supervision visits at regional and district level are often conducted in the context of training or for priority programmes. The district teams conducted integrated supervision visits only at community health centre level.

Civil service regulations state that performance appraisals should be conducted annually. Only 48% received this during the last two years. In the qualitative study, interviewed staff appeared unaware of the criteria used. One health worker said: "It is subjective, as the boss appraises according to his own criteria".

### Rewards

There were no formal methods in place in Mali to show appreciation and give rewards. A few managers congratulated and thanked personnel in public. Some assigned well-performing staff to supervision visits or training, to enable them to gain extra income from allowances. Managers did not seem to show appreciation; as one health worker said: " I feel that I do a good job. My boss appreciates me, but I do not know how. He does not say anything". Some health workers said they depended on the beneficiaries to feel appreciated, because patients thank the health workers and give them presents.

### Career development

Twenty-two percent of the respondents were not satisfied with their current career path. However, there is a large variation within regions: health workers in the cities were relatively satisfied, but 45% of the respondents in the remote areas were not. In total, 71% received a promotion in their working career, 48% of which was based on age and 26% resulting from training (mainly auxiliary nurses becoming registered nurses).

## Discussion

The study revealed that the main motivators for health workers in all eight professional categories were related to recognition or appreciation, responsibility and training. This corresponds with other studies on motivation of health workers in resource-poor settings [3,13,14]. Distinguishing between motivators and demotivators enables managers to concentrate on addressing those related to motivation (and consequently performance). The appropriateness of the current methods of improving staff performance (study question 3) was determined by analysing the match between the identified motivators against the performance management activities in use.

The implementation of various performance management activities in Mali could be improved upon. Some activities, such as promotion, career development and performance appraisal are mainly administrative rituals and not used to enhance performance. Job descriptions were not specific enough to allow the identification of training needs or to feel – or be held – responsible. Overall, performance management activities did not seem to be linked to each other. For instance, job descriptions did not seem to be linked to identifying training needs and to selection of health workers to participate in training. This is also found in other countries; a study among 15 organizations in various countries showed that integrated performance management systems were found in only three organizations [7].

Also, health workers did not seem to find the decisions of managers transparent: for instance, in training and performance appraisals. In addition, performance management could be better focused on achieving the purpose of health facilities, which is the provision of good, accessible care. Staff seemed reasonably happy with the continuous education and supervision opportunities. Yet training and supervision were usually based on the needs of centrally run priority programmes rather than broader local needs.

Despite the focus on motivators, the findings indicate that the lack of materials was an important demotivator. Such demotivators could be addressed by improved management. This shows that attention to broader management tasks is also needed to improve performance, as documented elsewhere [15]. Therefore, addressing HRM issues is necessary but not sufficient to improve performance. But the study revealed that management development is neglected in training and supervision.

The main motivating factors identified in this study – recognition, responsibility and training – seem to correspond with the satisfiers mentioned by Herzberg. However, this should be concluded with caution, as it was not always clear whether the motivation for training, for example, was really related to advancement by updating knowledge (satisfier) or to complementing a salary (dissatisfier). In addition, in our study, salary was seen as an important motivator, whereas Herzberg categorizes this as a dissatisfier. This could be due to how the questions were asked or to the fact that salaries among health workers in Mali were very low and thus earning sufficiently to provide for the family was the most important issue on health workers' minds.

## Conclusion

Although salaries and incentives are important factors for health workers and should not be neglected, the study does show that gains in motivation could be made by giving greater responsibility to staff, by holding staff responsible and by improving mechanisms for recognition. These gains in motivation, which would ultimately contribute to improving quality of care and accessibility, could be achieved through improved performance management activities matched to these motivating factors.

For managers, Herzberg's model could be a useful way of thinking about the two types of motivation and for selecting appropriate strategies to address them. The formulation of suitable HR activities, however, should be preceded by identifying which factors are motivating for health workers in their specific contexts.

As a result of the study, a recommendation was made to the Ministry of Health in Mali to adapt performance management strategies to the motivators that were identified – for example, by relating performance appraisal to tasks for which these health workers are responsible according to their job descriptions.

Other countries could also use operational research to identify the predominant motivators in order to adapt their performance management strategies, though care must be taken with asking questions about motivating factors, due to wide possibilities of interpretation of terms such as appreciation and recognition and the perceived benefits of activities such as training. Pretesting is required. The resulting set of HRM systems and tools may require a radical change in management culture, especially if they include more participatory decision-making and a problem-solving approach, enhancing trust-building between health workers and managers [16].

Priority programmes have an important contribution to make, by better aligning their performance management activities such as training and supervision with HRM activities of the managers at the existing facilities. They should also better coordinate and integrate their activities with those of other priority programmes and with health services plans, and develop explicit strategies to strengthen HRH management systems in the health sector. These actions would contribute to creating a more productive workforce that delivers quality of care.

## Competing interests

The author(s) declare that they have no competing interests.

## Authors' contributions

Marjolein Dieleman was a member of the research team and contributed to protocol development, data collection and analysis and writing the report of the study. She headed the team for the qualitative study component. She wrote and revised the manuscript.

Jurrien Toonen was a member of the research team and contributed to protocol development, data collection and analysis and writing the report of the study. He headed the team for the survey. He reviewed the manuscript.

Hamadassalia Toure was a member of the research team and contributed to protocol development, data collection and analysis and writing the report of the study. He reviewed the manuscript.

Tim Martineau assisted in preparation of the research and contributed to protocol development; he revised the manuscript.
